# Hypercapnia Induced by Pectus Excavatum and Cardiomegaly

**DOI:** 10.31662/jmaj.2023-0162

**Published:** 2024-04-01

**Authors:** Shota Shirotani, Yasuhiro Kano, Hiroyuki Tanaka

**Affiliations:** 1 Department of Cardiology, Tokyo Metropolitan Tama Medical Center, Tokyo, Japan; 2 Department of Emergency and General Medicine, Tokyo Metropolitan Tama Medical Center, Tokyo, Japan

**Keywords:** pectus excavatum, cardiomegaly, hypercapnia

An 89-year-old man was admitted with acute decompensated heart failure, which was possibly due to nonischemic cardiomyopathy. Upon admission, arterial blood gas analysis demonstrated acute hypercapnia with pH 7.17, PCO_2_ 91.9 mm Hg, and HCO_3_ 33.6 mmol/L. Transthoracic echocardiography revealed that the left ventricular, end-diastolic diameter had increased to 55 mm. Computed tomography demonstrated heart enlargement and its dislocation leftward by the pectus excavatum, causing almost complete occupancy of the left thoracic cavity by the heart and a disturbance in left lung expansion (Figure 1). His hypercapnia resolved with intravenous furosemide and noninvasive positive pressure ventilation along with cardiomegaly improvement. Hypercapnia caused by the lung expansion restriction by the pectus excavatum and cardiomegaly was finally diagnosed.

**Figure 1.**
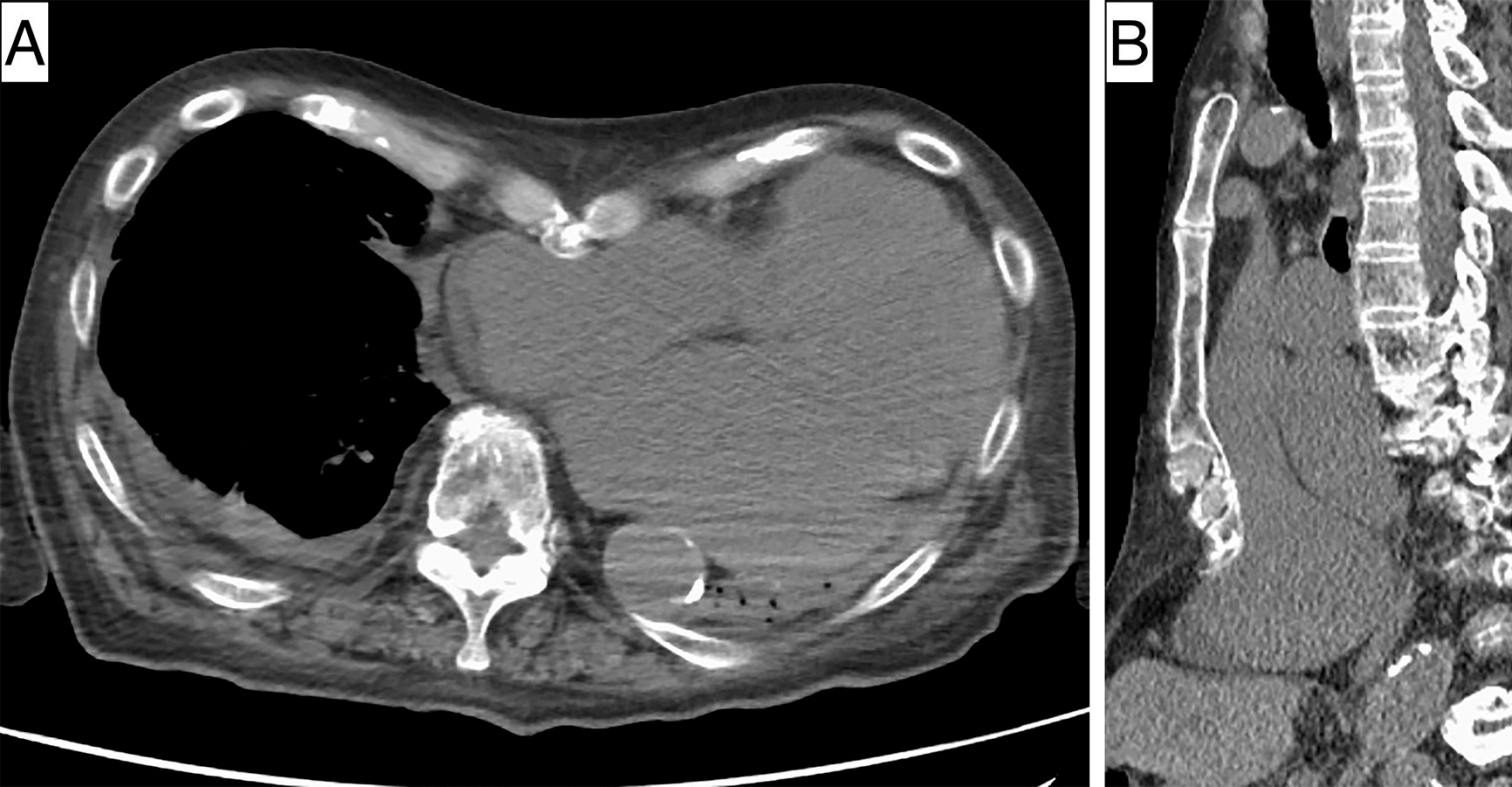
Chest computed tomography showing that the patient’s enlarged heart was pushed leftward by the narrowed chest cavity caused by the pectus excavatum, and his heart “wedged” into the left cavity, causing almost complete occupancy of the left thoracic cavity by the heart and a disturbance in left lung expansion.

Pectus excavatum is a common deformation caused by depression of the anterior chest wall into the thoracic cavity. Although it is usually asymptomatic, severe cardiomegaly associated with this condition can decrease forced vital capacity by disrupting lung expansion ^(1), (2)^, potentially leading to a life-threatening hypercapnia.

## Article Information

### Conflicts of Interest

None

### Acknowledgement

The authors thank Mr. James R. Valera for his assistance with editing this manuscript.

### Author Contributions

Shota Shirotani, Yasuhiro Kano, and Hiroyuki Tanaka were involved in the conception or design of the work, drafting the work or reviewing it critically for important intellectual content, final approval of the version to be published, and agreement to be accountable for all aspects of the work in ensuring that questions related to the accuracy or integrity of any part of the work are appropriately investigated and resolved.

### Informed Consent

Consent to publish the details of the present case was obtained from the patient.
